# The Role of Nutrition in the COVID-19 Pandemic

**DOI:** 10.3390/nu13041093

**Published:** 2021-03-27

**Authors:** Maria Chiara Mentella, Franco Scaldaferri, Antonio Gasbarrini, Giacinto Abele Donato Miggiano

**Affiliations:** 1 UOC di Nutrizione Clinica, Fondazione Policlinico Universitario A. Gemelli IRCCS, Università Cattolica del Sacro Cuore, 00168 Rome, Italy; giacintoabele.miggiano@unicatt.it; 2 UOC di Medicina Interna e Gastroenterologia, Fondazione Policlinico Universitario A. Gemelli IRCCS, Università Cattolica del Sacro Cuore, 00168 Rome, Italy; franco.scaldaferri@policlinicogemelli.it (F.S.); antonio.gasbarrini@unicatt.it (A.G.)

**Keywords:** COVID-19, SARS-CoV-2, nutritional status, malnutrition, obesity, undernutrition, nutrients deficiencies, older adults

## Abstract

SARS-CoV-2, the cause of the COVID-19 disease, is posing unprecedent challenges. In the literature, increasing evidence highlights how malnutrition negatively affects the immune system functionality, impairing protection from infections. The current review aims to summarize the complex relationship between SARS-CoV-2 infection and nutritional status and the effects of malnutrition in terms of disease severity, patients’ recovery time, incidence of complications and mortality rate. Current studies evaluating the possibility of modulating nutrition and supplementation in combination with pharmacological treatments in the clinical setting to prevent, support, and overcome infection are also described. The discussion of the most recent pertinent literature aims to lay the foundations for making reasonable assumptions and evaluations for a nutritional “best practice” against COVID-19 pandemic and for the definition of sound cost-effective strategies to assist healthcare systems in managing patients and individuals in their recovery from COVID-19.

## 1. Introduction

The contemporary severe acute respiratory syndrome coronavirus 2 (SARS-CoV-2), responsible for coronavirus disease 2019 (COVID-19), was first observed in December 2019 in the city of Wuhan, China [1]. Then, the fast diffusion of the disease led the World Health Organization (WHO) to declare a status of international health emergency, considering the effects the virus could have all over the world and in particular in underdeveloped countries with lower-quality health infrastructure [2]. The COVID-19 outbreak has deeply changed human life and brought new challenges to worldwide healthcare systems, which are currently allocating significant efforts in the development of vaccines, in the identification of therapeutic solutions and in the containment of the infection through restrictive measures such as social quarantine. Clinical manifestations of SARS-CoV-2 range from asymptomatic infection to the onset of serious pneumonia, acute respiratory syndrome, acidosis, coagulation dysfunction, organ failure, and death [3,4]. In such a multifaceted spectrum of clinical manifestations, it is crucial to identify predictive factors associated with mild, severe, or critical outcomes.

## 2. Methodology

A literature search was performed between January 10 and February 10, 2021, on PubMed database using search terms such as: SARS-CoV-2, COVID-19, nutritional status, older adults, obesity, malnutrition, undernutrition, nutrients deficiency, supplementation. All types of articles related to humans only were included for evaluation. Articles for which full text was not available and articles which were not in English were excluded. From the articles retrieved in the first round of searches, additional references were identified by a manual search among the cited references. Studies were critically appraised, and the findings were analyzed. A summary of the included studies is reported in Table 1. A narrative approach was used to summarize and present the results.

## 3. Malnutrition in the COVID-19 Pandemic

The retrospective assessment of data of the Spanish flu in 1918, which killed an estimated 50 million victims worldwide, suggested that disease severity resulted from an articulated interaction between viral, societal and individual factors [29]. According to recent clinical evidence in the COVID-19 pandemic scenario, several aspects have been correlated with more critical patients’ admissions to hospital, higher rate of complications, longer recovery time, and even higher mortality rate. In particular, recent studies have reported how malnutrition is one of the crucial elements that may be predictive of slower recovery, or no recovery at all, for the affected subjects [30]. Malnutrition refers to the incorrect intake of both energy and macronutrients (carbohydrates, proteins, fats), as well as to micronutrient (minerals and vitamins) deficiency. In the case of shortage of energy intake, the food energy fails to meet the individual’s needs, whereas micronutrient deficiency refers to a lack of vitamins and minerals which are needed, in small amounts, for healthy growth and development. Although it might seem contradictory, individuals might be overfed in terms of energy but be deficient in one or more micronutrients in their routine diet. Inadequate intake of these nutrients is currently largely diffuse, leading to an impaired resistance to infections and, consequently, to an increase in disease seriousness [31].

In January 2016, the major clinical nutrition societies organized the Global Leadership Initiative on Malnutrition (GLIM) to agree on shared key criteria for the classification of malnutrition in adult people in clinical contexts [32]. These allow defining malnutrition by first screening for malnutrition risk, and then assessing for diagnosis and severity classification. The main criteria involve assessing three parameters concerning clinical manifestation (reduced body mass index, involuntary weight loss, and low muscle mass) and two etiologic parameters (low food intake or assimilation, inflammation or any burden related to the disease). Malnutrition is diagnosed when at least one clinical manifestation and one etiology criterion are present. Malnutrition may be staged as moderate (Stage 1) or severe (Stage 2) according to phenotypic parameters, and fall within one of four categories concerning its etiology, by considering if it is caused by a chronic disease, distinguishing if inflammation is either present or absent, by an acute inflammatory disease, or by starvation (even when related to socio/economic or environmental causes implying food shortage or hunger).

### Malnutrition, COVID-19 Infection, and the Immune System

Nutrition is pivotal in supporting the immune system. Immune homeostasis is indeed well-regulated by a balanced nutrition. Calder et al. reported that an adequate nutrition regimen is key in the defense against viral threats [31]. In the current scenario, in which the changes in dietary and lifestyles habits, largely due to social distancing, might have significantly contributed to a deprived nutritional status, immune system functionality might be undermined. The immune response could indeed be impaired even by minor deficiencies or insufficiencies of some micronutrients [33,34]. Importantly, this can be reversed by correcting the patient’s nutritional status.

The European Food Safety Authority (EFSA) scientific panel has highlighted how the healthy maintenance of the immune system strictly depends upon vitamins D, C, A (including β-carotene), and those of group B (particularly B6, B12 as well as folate). Zinc, copper, iron, and selenium are given similar roles. Taking this into account, Galmés et al. [35] have published an updated report on the relevance of nutrition as an immune-enhancing factor. Results from their review demonstrate the importance of preserving a well-balanced level of these ten nutrients, emphasizing the key role played by vitamin D as well as iron as far as the current pandemic is concerned. Relevant micronutrient intake levels—especially those of iron and vitamins B12, C and D—have been found to present an inverse correlation with higher disease incidence and fatality rate, especially in populations showing genetic predisposition to poorer micronutrient status. On the other hand, the wide prevalence of malnutrition and trace element deficiency all over the world will likely affect the global COVID-19 outcomes [36]. 

## 4. COVID-19 and Obesity 

Although knowledge about the nutritional profile of patients suffering from COVID-is still limited, the currently available evidence shows that nutritional disorders are linked to worse clinical outcomes, as well as with increased infection risk. In a retrospective study [5] involving a cohort of 210 SARS-CoV-2 patients aged 18–45 years, investigating possible relationships between obesity (defined as BMI > 30 kg/m^2^) in-hospital mortality, and need for mechanical ventilation, obesity was found to be an independent and strong factor increasing the risk of negative outcomes. In detail, obesity was significantly associated with mortality (Odds Ratio (OR), 6.29; 95% confidence interval (CI), 1.76–22.46), the need for mechanical ventilation (OR, 6.01; 95% CI, 2.5–14.48) and hospital admission (OR, 2.61; CI, 1.49–4.58) (*p* < 0.05 in all cases). 

Simmonet et al. [6] recently reported how around 90% of patients admitted to intensive care units (ICU) having a BMI >35 kg/m^2^ needed mechanical ventilation. The authors concluded that obesity is a strong negative prognostic risk factor for COVID-19. Obesity may modulate the risk for infection as well as the overall clinical outcomes in affected patients, as it was observed for the H1N1 swine flu during the 2009 pandemic [7]. This could be in large part explained by an altered, macrophage-mediated, inflammation state of the adipose tissue. 

In the COVID-19 scenario, patients present an amplified and critical inflammatory response known as a “cytokine storm”, due to activation of macrophages, the most represented immune cells within the adipose tissue. Evidence concerning how inflammation develops in COVID-19 obese patients is currently scarce. Moreover, clinical findings reported for H1N1 flu might also be considered valid in the COVID scenario; indeed, in flu patients, inflammatory marker levels, including those of TNF-a, IL-6, IL-8, and IL-15, were associated with poorer clinical outcomes and were found to be higher in obese patients than in lean ones [8].

In a retrospective study [9] assessing the relationship between the distribution of adipose tissue and symptoms severity in 143 patients hospitalized with confirmed COVID-19, risk factors that were found to be independently and significantly correlated with critical symptoms were high visceral adiposity (the ratio between visceral to subcutaneous tissue area (OR, 2.47; CI, 1.05–5.98, *p* < 0.05) and high intramuscular fat deposition (i.e., the skeletal muscle showing low mean attenuation (OR, 11.90; CI, 4.50–36.14; *p* < 0.001). Both quantities were also found to be predictive risk factors for mechanical ventilation (high visceral adiposity, *p* = 0.013; high intramuscular fat deposition, *p* < 0.001), the latter also increasing the risk of death (*p* = 0.012). The authors conclude that both high visceral adiposity and intramuscular factor are negative prognostic factors for critical COVID-19 progression. 

A retrospective study involving 150 consecutively enrolled patients affected by COVID-19 and admitted to an emergency department in Rome, Italy, [10] provided similar results. Patients requiring intensive care had significantly higher visceral fat (*p* = 0.032), with other significant factors being age (*p* = 0.009), C-reactive protein (CRP) (*p* < 0.0001), lactate dehydrogenase (LDH) (*p* = 0.003) and a high lung severity score (LSS) (*p* < 0.0001), indicating the patient suffering from severe interstitial pneumonia. All these factors, as well as increasing lymphocytes, D-dimers, visceral adipose tissue (VAT), and abdominal fat, correlated significantly with the patient needing intensive care, with LSS and VAT being independent between each other (OR, 1.262; CI, 1.0171–1.488; *p* = 0.005 for LSS and OR, 2.474; CI, 1.017–6.019; *p* = 0.046 for VAT). Thus, VAT was confirmed as a negative prognostic factor for the disease progression.

These results taken together suggest that obesity is a negative prognostic risk factor in COVID-19 disease progression, its effect also being independent from the patients’ age or gender, or the presence of comorbidities. Community efforts should focus on reducing the prevalence of overweight and obesity, and the promotion of healthy eating habits and regular physical activity.

## 5. COVID-19 and Undernutrition

Undernutrition, defined as a pathologic condition involving the nutritional regimen failing to reach the individual’s nutritional or energy needs, may derive from an unbalanced intake of micronutrients or macronutrients, from an excessive daily energy expenditure, from impaired nutrient absorption, or from any of the abovementioned possibilities combined together [37]. In this scenario, either protein–energy malnutrition, or deficiencies in some nutrients, are associated to the enhanced risk of most frequently occurring contagious diseases [38]. Li et al. [11] highlighted, in a cross-sectional study, a 52.7% prevalence of undernutrition in elderly patients. Malnutrition has been identified as a negative prognostic factor, being correlated to increased hospital length of stay, death, and re-admission rates [12]. 

In a monocentric, retrospective trial carried out in Wenzhou, China, involving 122 COVID-19 patients hospitalized between January and February 2020, a poorer nutritional status was found to predispose patients to a severe manifestation of COVID-19 infection [13]. A total of 105 patients (86.1%) had a common COVID-19 form; 17 cases (13.9%) had a serious one. They had their Prognostic Nutritional Index (PNI) values calculated as 5 × total lymphocyte count (/nL) + serum albumin (g/L). Patients suffering from the severe COVID-19 form has had a significantly smaller PNI (*p* = 0.029), independent of their BMI, gender, or age range. PNI scores showed an independent and inverse association with COVID-19 severity (OR: 0.797; *p* = 0.030), after adjusting for confounding factors such as demographics, indexes for liver and renal functionality, C-reactive protein levels, and smoking habits.

According to the publication of Anker et al. [14], which summarizes data from three study reports for a total of 589 cases, COVID-19 patients likely undergo significant weight loss, up to developing cachexia (weight loss ≥ 5%), a condition that affects both the muscle and fat tissue. The mean frequency of cachexia was 37% (range 29–52%). According to the authors, several nutritional and metabolic aspects such as increased body temperature, reduced appetite, and organ-specific complications involving the heart and kidneys can contribute to weight loss linked to COVID-19. 

In an longitudinal study, involving all patients admitted to a major French hospital from March to April 2020 [15], the overall prevalence of malnutrition was 42.1% (moderate in 23.7% of the cases and severe in 18.4%). In this study, the nutritional status was defined using the GLIM criteria. A majority (66.7%) of patients that had been in an ICU presented with malnutrition. The authors, however, did not assess muscular strength, which is one of the manifestation GLIM criteria for malnutrition [32]. Lower albumin levels were a significant risk factor for admission to an ICU (OR, 0.31; CI, 0.1–0.7 for any 10 g/L albumin; *p* < 0.01), independent of age and CRP values.The NUTRICOV prospective observational cohort study, conducted by Rouget et al. over the same period [16] on 80 COVID-19 patients, showed that 37.5% of them had malnutrition based on GLIM criteria.

In a major South Korea center, thirty-eight (76%) out fifty adults suffering from COVID-19 were found to present low vitamin D levels, and 21 (42%) had low selenium values [17]. Severely low vitamin D values (≤10 ng/dL) were found in about 24% of the COVID-19 patients, compared to 7.3% in the control group. Eleven (91.7%) of 12 patients with respiratory distress were deficient for at least one nutrient. Furthermore, 78.9% of patients without respiratory distress were classified as nutrient-deficient (*p* = 0.425). These results taken together showed a high prevalence of vitamin D deficiency; moreover, all critically ill cases were deficient in more than one nutrient.

The Controlling Nutritional Status (CONUT), an index calculated on the base of lymphocyte count, total cholesterol, and serum albumin, was found to be a further risk factor for the death of COVID-19 patients [18]. Low serum albumin values alone also seem associated with the disease severity [19]. Data concerning undernutrition and COVID-19 are still limited; thus, it is not possible to draw robust conclusions on a possible relationship between these conditions. However, considering the known inflammatory response caused by this undernutrition [39], it is likely to expect it to be associated with an impaired immune response and higher risk of complications, as found in obese patients [40]. Targeted indications for an adequate nutritional approach in the treatment strategy within the pandemic scenario have indeed been outlined [41].

Malnutrition and undernutrition, therefore, are conditions potentially worsening the disease severity and outcome; however, symmetrically, suffering from COVID-19 creates a condition leading to body weight loss and malnutrition [41]. In particular, a wide range of symptoms of COVID-19 can negatively interfere with dietary behavior. As reported by Holdoway [42], common symptoms of COVID-19 infection that can affect food intake include:

- Breathing difficulties that might limit what patients can eat or drink, such as cough, and shortness of breath; air trapping or early satiety, caused by gulping for air while swallowing and dry mouth due to the impaired nasal breathing, use of inhalers and oxygen therapy;
- Smell or taste loss which can decrease appetite and desire to eat food;
- Increased body temperature which boosts nutritional needs and inflammatory response, reduces appetite, and contributes to muscle loss;
- Feeling of tiredness, which impairs patient’s ability to carry out normal daily activities.

Additionally, physical distancing and isolation may reduce mealtime care, and social interactions with other people which often concur to healthy food consumptions.

On the whole, the findings reported above stress the need for assessing the overall nutritional status of patients particularly at the first clinical evaluation as well as at the hospital admission and do highlight the importance of identifying the nutrition-related factors that could affect disease evolution and prognosis.

### COVID-19, Undernutrition, and Older Adults

As far as COVID-19 outcomes in older people are concerned, it has rapidly become clear that older persons have a high likelihood to be infected by SARS-CoV-2, and that the infection seems to be particularly lethal in this subgroup of patients [43]. Not surprisingly, persons living in nursing homes show a high risk of contracting, as well as dying from, COVID-19 [44]. With reference to this subpopulation, Recinella et al. [20] found, in a monocentric study on 109 patients, that older persons hospitalized for COVID-19 and at high nutritional risk, as determined by the Geriatric Nutritional Risk Index, had an increased risk of mortality. They also observed that older persons with hypoalbuminemia and a low BMI had an increased death rate. With reference to older people, Pironi et al. [21], applying GLIM criteria, found that 50% of COVID-19 hospitalized patients (70% of included patients were older than 65) were malnourished. In one study conducted in Wuhan, China, Li et al. used the MNA (Mini Nutritional Assessment) questionnaire and reported that 52.7% of over 65-year-old hospitalized COVID-19 patients suffered from malnutrition, and 27.5% of them presented risk of malnourishment [11]. A similar trial, again using the MNA as a measuring tool, reported that 65.9% of COVID-19 patients, aged 55 years on average, were at risk of undernutrition and 14.6% were undernourished [22].

Liu et al. [23] compared the Malnutrition Universal Screening Tool (MUST), the Nutritional Risk Screening 2002 (NRS 2002), the Nutritional Risk Index (NRI) and the MNA Short Form (MNA-SF) in hospitalized subjects with COVID-19-infection above the age of 65. According to the authors, MNA-SF, NRI, and NRS 2002 enabled the identification of patients at need of prolonged hospitalization and showing poor appetite, higher disease severity, and higher weight loss when compared with patients with normal nutritional status. 

**Figure 1:**
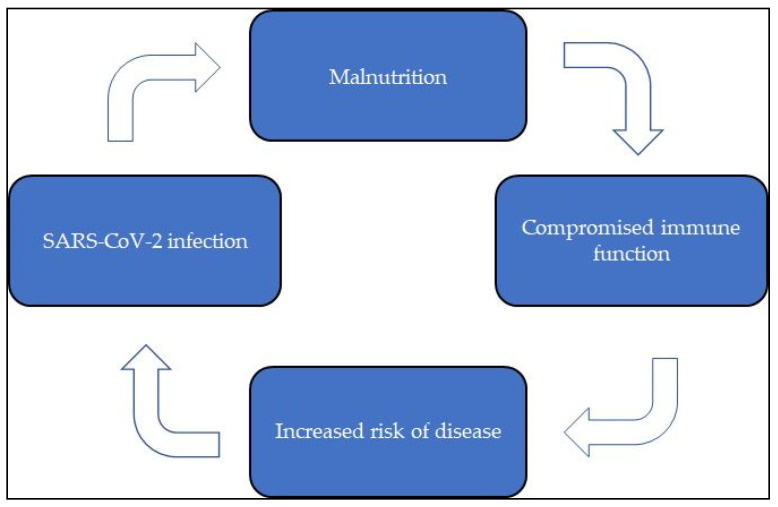
The vicious circle connecting malnutrition to immune system impaired activity and individual SARS-CoV-2 infection susceptibility.

These results also stress the importance of nutritional support in COVID-19 care, as emphasized by the European Society for Clinical Nutrition and Metabolism (ESPEN) [41] and other clinical experts [45,46]. The data collected and discussed so far reinforce the evidence of a fil rouge connecting malnutrition to immune system impaired activity and susceptibility to SARS-CoV-2 infection in a vicious circle, as schematized in the following figure (Figure 1).

In this scenario, the international recommendations concerning nutrition in the ICU [41] should be properly applied. Based on these indications, Thibault et al. [47] proposed a nutrition protocol for COVID-19 patients, whose key aspects are summarized below:

1. Nutritional status of COVID-19-affected individuals needs to be carefully evaluated;
2. Nutritional evaluation performed according to the GLIM criteria needs to be adjusted to the current pandemic scenario;
3. To avoid overfeeding, one should consider using indirect calorimetry (IC) only for patients who are unstable and in the ICU for > 10 days, or those on complete parenteral nutrition (PN);
4. Propofol administration may cause complications that must be avoided; refeeding syndrome (RS) must be avoided;
5. PN should be not preferred to enteral nutrition (EN), and this should begin within two days from admission;
6. Generally, gastric EN is feasible. It can also be performed with the patient in a prone position. Pumps with flow regulation should be preferred;
7. PN should be considered if EN is either not possible, not indicated or no sufficient, and only after individual case assessment;
8. Omega-3 fatty acid-enriched EN is the choice of preference when acute respiratory distress syndrome is present. If PN is necessary, prescription of intravenous fat emulsions enriched with fish oils is advised;
9. Nutrition therapy should be maintained as long as necessary to allow the patient regaining sufficient oral intake after extubation;
10. Muscle reserves and functionality should be preserved by promoting mobilization.

In conclusion, considered the results reported above, nutritional therapy appears to be key in the management of COVID-19-affected patients and should be properly implemented in the standard clinical practice.

## 6. The Benefits of Supplementation in the COVID-19 Pandemic

The current evidence on the infection suggests that it causes a generalized status of enhanced inflammation due to the release of a “cytokine storm”. The patient is hospitalized and, if needed, admitted to an ICU to enable overcoming the “inflammatory storm”. According to Ferrara et al. [48], combining anti-inflammatory and antiviral drug treatment seems effective in inhibiting the release of cytokines, for the prevention of lung collapse, and for a radical reduction in the SARS-Cov-2-related death rate. 

Combining pharmacological treatments with nutritional interventions and amino acid supplementation with the aim of preventing, supporting, and overcoming the infection is under evaluation. The hypothesis is that the association of clinical nutrition and adequate supplementation might support the immune system, enhancing its preventive action against infection and ensuring that inflammation is controlled. Above all, amino acids seem to provide a relevant contribution to those affected by the disease and hospitalized in an ICU: on one side, it regulates inflammation; and on the other, it promotes the healing phase with consequent indirect and intangible cost savings [46,49].

The utilization of probiotics (i.e., microorganisms that, when administered in adequate amounts, provide a health benefit on the host) is also under evaluation, which is recognized to be useful for improving immunity. Probiotics, in fact, modulate the activity of host immune cells in the intestinal epithelium and mucosa [50,51]. Nevertheless, in order to obtain the most appropriate immunomodulatory/stimulatory effects, probiotics should be rationally selected among mono-strain, multi-strain and multi-species, considering the specific disease [24]. In particular, probiotics containing Lactobacillus may help prevent viral infections such as influenza, as reported by Berggren et al. [25] in a, parallel, double-blind randomized controlled study.

Considering the current pandemic scenario, Zuo et al. [26] analyzed 15 fecal samples from as many COVID-19 cases through shotgun metagenomic sequencing analyses, and observed notable alterations in their microbiomes compared to controls, with an increased presence of opportunistic and pathogenic bacteria and depleted commensal flora, at the hospital entrance and during the whole stay. Although an unreasoned intake of probiotics against COVID-19 may not be recommended until the SARS-CoV-2 pathogenesis and its consequences on the gut microbiota will become clearer, it is possible that a targeted approach aimed at modulating the gut microbiota will soon become a possible additional or adjuvant treatment option to manage the disease and its related comorbidities. Several clinical investigations are currently being undertaken to study the safety and effectiveness of probiotics in the current pandemic scenario, as summarized by Baindara et al. [52].

As reported by Pecora et al. [53], in vitro tests and observational studies highlight the importance of omega-3 fatty acids, vitamins A, C, D, and zinc for immune response modulation. Supplementing them within the context of a well-balanced diet and not exceeding the quantity limits defined by medical experts to support immune system functionality seems a low-priced and safe option, potentially helping to prevent the infection, or to limit its consequences. 

With regard to omega-3 fatty acids, Vivar-Sierra et al. [54], in an in silico simulation study, found that fatty acids high in omega-3 from a marine origin are correlated with lower COVID-19 mortality rates, and that those molecules could contribute to the reduction in COVID-19 medical complications by reducing virus entry into human cells, through a mechanism involving fatty acids binding to viral spike proteins. 

Hathaway et al. [55] hypothesized that supplementing omega-3 fatty acids might add to the prevention of the virus entry by changing the lipid composition in the bilipid membrane of cells. Moreover, omega-3 is key in mediating inflammatory processes, modulating both innate and acquired immune responses. However, future research should be carried out to clarify whether the supplementation of omega-3 fatty acids may play a pivotal role in SARS-CoV-2 treatment.

Recent published evidence consistently shows a strong association between vitamin D deficiency and severe COVID-19 manifestations [56]. Moreover, current hypotheses on the role played by vitamin D in immunity do provide support for an increasing number of clinical trials that are being undertaken to investigate how supplementing vitamin D may affect the COVID-19 outcomes [56]. Until these studies provide further evidence, guaranteeing sufficient vitamin D levels seems to be a reasonable and conscious measure. 

Interestingly, a controlled, double-blind, parallel arm randomized clinical trial is currently ongoing to evaluate if supplementing curcumin–piperine may be effective to manage the COVID-19 infection severity, duration, clinical manifestations and inflammatory mediators [27]. Similarly, a controlled, double-blind, parallel arm randomized trial will study the effects of propolis supplementation [28].

Future results from these trials will shed light on possible benefits of these options on COVID-19 infection. Existing clinical evidence regarding the possible effects of supplementation on COVID-19 infection is still limited. Most of the investigations, to date, are retrospective, and observational; thus, data should be interpreted with caution. However, medical nutrition therapy is one of the pillars of COVID-19 management and represents a key tool in the panorama of the possible therapeutic interventions [48]. As discussed, malnourished people with impaired immune response and chronic diseases present a worse prognosis and higher fatality rates. Healthy nutrition not only supports the immune systems reaction against diseases, including COVID-19, but it also represents undoubted support promoting recovery from the disease.

## 7. Conclusions

Here, we discussed the relevance of nutritional status in COVID-19 patients, confirming the existing relationship between nutrition status, immune response, and disease clinical manifestations severity. In COVID-19, this relationship has shown to be crucial across the disease phases, particularly in people at risk for a poor prognosis, including obese, undernourished, and older patients. Malnutrition is largely recognized to be both a cause and a consequence of immune system dysfunction. Even prolonged stay in an intensive care unit is a known risk factor for malnutrition, often causing dramatic muscle mass loss and physical function impairment. The altered inflammatory response consequent to the SARS-CoV-2 infection may aggravate catabolic processes and cachexia. These aspects may, in turn, worsen malnutrition and contribute to slow recovery, loss of independence in the daily life, depression, disability, and generally to a decreased quality of life after ICU discharge.

According to ESPEN recommendations, programs addressing the care of COVID-19 patients should integrate nutritional screening, assessment, and therapy. Obesity is a negative prognostic risk factor in COVID-19 disease progression, its effect also being independent of patient age or gender, or the presence of comorbidities. Underfeeding in individuals with COVID-19 should be carefully avoided, and ad hoc nutrition protocols and practical indications should be largely adopted in hospitals. Moreover, research will soon shed light on the possible benefits that ad hoc nutrient supplementation may provide to patients. In this pandemic scenario, community efforts should focus on promoting healthy eating and active living habits, thus preventing overweight, obesity or nutrient deficiency. In this respect, following guidelines for a healthy diet is highly recommended to prevent malnutrition in all its forms. Healthcare services should define and implement cost-effective strategies focused on increasing population awareness on the importance of balanced nutrition regimens, also considering the no imminent end of pandemic.

A multidisciplinary approach is likely to bring the highest benefit to patients. Unfortunately, most of the current knowledge is based on evidence that is mainly retrospective and observational or on previous investigations concerning other populations affected by infectious diseases; thus, it is not possible to draw robust conclusions and further recommendations. Future research should focus on investigating how malnutrition relates to the COVID-19 course and prognosis through rigorous, ad hoc clinical studies.

## Figures and Tables

**Table 1:** Summary of the included articles.

Publication	Country	Temporary Frame (Beginning of the Pandemic, Middle, Second Wave)	Type of Study	Number of Centers	Number of Patients	Type of Patients (Asymptomatic, Infected, Hospitalized, Admitted in Intensive Care Unit)	Exposure of Interest (BMI/Energy Shortage, Micronutrients Deficiency)	Key Findings of the Study	Study Conclusions
Chen et al., *Lancet*, 2020 [3]	China	Beginning of the pandemic	Retrospective study	Monocentric	99	Infected	NA	COVID-19 patients present clinical manifestations of fever, cough, shortness of breath, muscle ache, confusion, headache, sore throat, rhinorrhea, chest pain, diarrhea, and nausea and vomiting	The infection of 2019-nCoV was of clustering onset, is more likely to infect older men with comorbidities, and can result in severe and even fatal respiratory
Steinberg et al., *BRR*, 2020 [5]	U.S.A.	Beginning of the pandemic	Retrospective cohort study	2	210	Infected	Obesity (BMI > 30 kg/m^2^)	Association between primary outcomes (in-hospital mortality, need for IMV, and admission to hospital) and obesity	Obesity appears to be an independent risk factor for poor outcomes in young patients with COVID-19
Simmonet et al., *Obesity*, 2020 [6]	France	Beginning of the pandemic	Retrospective cohort study	Monocentric	124	Admitted to ICU	Obesity and severe obesity (BMI > 35 kg/m^2^)	The proportion of patients who required IMV increased with BMI categories	Obesity is considered a risk factor for SARS-CoV-2 severity
Louie et al., *CID*, 2011 [7]	U.S.A.	NA	-	Monocentric	534	Hospitalized	Obesity (BMI ≥ 30 kg/m^2^) and extreme obesity (BMI ≥ 40 kg/m^2^)	BMI > 40 kg/m^2^ is an independent risk factor in hospitalized adults for death from 2009 H1N1 infection	Extreme obesity was associated with increased odds of death
Hagau et al., *CC*, 2010 [8]	Romania	NA	Prospective study	Monocentric	32	Hospitalized	Obesity	In obese patients with influenza disease, a significantly increased level of IL-8 was found	Ill patients with nvA (H1N1) virus infection have increased levels of some cytokines
Yang et al., *Obesity*, 2020 [9]	China	Beginning of the pandemic	Retrospective study	Monocentric	143	Hospitalized	-	Association between adipose tissue distribution and severity of clinical course	COVID-19 patients with visceral adiposity or high IMF deposition have higher risk for critical illness
Watanabe et al., *Metabolism*, 2020 [10]	Italy	Beginning of the pandemic	Retrospective study	Monocentric	150	Infected	Obesity	Visceral fat (VAT) was significantly higher in patients requiring intensive care	VAT is a marker of worse clinical outcomes in patients with COVID-19
Li et al., *EJCN*, 2020 [11]	China	Beginning of the pandemic	Cross-sectional study	Monocentric	182	Hospitalized	Risk of malnutrition and malnutrition	The prevalence of malnutrition in elderly patients with COVID-19 was high	Malnutrition has been identified as a negative prognostic factor
Agarwal et al., *CN*, 2013 [12]	Australia	NA	Prospective cohort study	56	3122	Hospitalized	Malnutrition	Malnourished patients had greater median LOS, readmissions rates and in-hospital mortality	Malnutrition and poor food intake are independently associated with in-hospital mortality
Hu et al., *Nutrition*, 2020 [13]	China	Beginning of the pandemic	Retrospective trial	Monocentric	122	Hospitalized	Undernutrition	Association between the prognostic nutritional index (PNI) score and the severity of COVID-19.	Poorer nutritional status predisposed patients infected with COVID-19 to its severe form
Anker et al., *JCSM*, 2020 [14]	Italy, France	Middle	Retrospective studies	-	589	Hospitalized	Risk of malnutrition and malnutrition	Patients with COVID-19 disease are prone to develop significant weight loss and clinical cachexia	Many metabolic and nutritional factors can contribute to body wasting in COVID-19
Bedock et al., *CN*, 2020 [15]	France	Beginning of the pandemic	Longitudinal study	Monocentric	114	Hospitalized	Moderate and severe malnutrition	Association between malnutrition and unfavorable outcomes (transfer to ICU or death)	Low albumin levels at admission are a predictive marker of more severe outcome of the disease
Rouget et al., *BRJN*, 2020 [16]	France	Beginning of the pandemic	Prospective observational cohort study	Monocentric	80	Hospitalized	Malnutrition	High prevalence of malnutrition in a general cohort of COVID-19 inpatients according to GLIM criteria	Nutritional support in COVID-19 care is an essential element
Im et al., *IJID*, 2020 [17]	South Korea	Middle	-	Monocentric	50	Hospitalized	Micronutrient deficiency	Vitamin D and selenium deficiencies were the most prevalent among COVID-19 patients	Deficiency of vitamin D or selenium may decrease the immune defenses against COVID-19 and cause progression to severe disease
Du et al., *Medxiv*, 2020 [18]	China	Beginning of the pandemic	Retrospective cohort study	Monocentric	245	Hospitalized	Malnutrition	In-hospital mortality was significantly higher in the severe group of PNI and in the severe-CONUT group	The CONUT score and PNI could be a reliable prognostic marker of all-cause death in patients with COVID-19.
Wu et al., JAMA, 2020 [19]	China	Beginning of the pandemic	Retrospective cohort study	Monocentric	201	Hospitalized	Malnutrition	The risk factors related to the development of ARDS and progression from ARDS to death included older age, neutrophilia, and organ and coagulation dysfunction	Older age was associated with greater risk of development of ARDS and death likely owing to less rigorous immune response
Recinella et al., *ACER*, 2020 [20]	Italy	Beginning of the pandemic	-	Monocentric	109	Hospitalized	Age > 65, malnutrition	Lower values of body weight, BMI, GNRI and albumin were found in patients experiencing in-hospital death. Higher values of GNRI were found in surviving patients	Nutritional status assessed by GNRI is a significative predictor of survival in elderly patients hospitalized for COVID-19
Pironi et al., *CN*, 2020 [21]	Italy	Beginning of the pandemic	Cross-sectional study	Monocentric	268	Hospitalized	Age, malnutrition	Very high prevalence of nutritional risk and malnutrition in adult patients hospitalized for COVID-19	The patient energy and protein intake were at the lowest limit or below the recommended amounts, indicating the need for actions to improve the nutritional care practice
Haraj et al., *CN_ESPEN*, 2020 [22]	Morocco	Middle	Descriptive observational study	Monocentric	41	Admitted in intensive care unit	Risk of malnutrition and malnutrition	Most COVID-19 patients were at risk of undernutrition	The nutritional diagnosis and the early nutritional management of COVID-19 patients must be integrated into the overall therapeutic strategy
Liu et al., *EJCN*, 2020 [23]	China	Beginning of the pandemic	Retrospective cohort study	Monocentric	141	Hospitalized	Age > 65, risk of malnutrition	The most of COVID-19 patients were at risk of malnutrition with longer LOS, higher hospital expense and worse disease severity	The NRS 2002, MNA-sf, and NRI are useful and practical tools with respect to screening for patients with COVID-19 who are at nutritional risk
Lin et al., *Vaccine*, 2009 [24]	Taiwan	NA	Double-blind, randomized, controlled study	4	1062	NA	Age < 5	*L. casei rhamnosus* can control bacterial, viral and respiratory infections	Bio-therapeutic agents may be useful in preventing viral and bacterial infectious disease
Berggren et al., *EJN*, 2010 [25]	Sweden	NA	Randomized, parallel, double-blind placebo-controlled study	2	272	NA	-	Treatments with probiotics mixtures shorten the duration, reduce the incidence of infection and/or lessen the severity of symptoms	Intake of probiotic mixture contributes to the body’s defense against common cold infections
Zuo et al., *Gastroenterology*, 2020 [26]	China	Beginning of the pandemic	Prospective study	Monocentric	36	Hospitalized	Altered intestinal microbiota	Patients with COVID-19 had significant alterations in fecal microbiomes compared with controls (enrichment of opportunistic pathogens and depletion of beneficial commensals)	Fecal microbiota alterations were associated with fecal levels of SARS-CoV-2 and COVID-19 severity. Strategies to alter the intestinal microbiota might reduce disease severity
Miryan et al., *Trial_pepper_curcumin*, 2020 [27]	Iran	Middle-ongoing (ending April 2021)	Randomized, placebo-controlled, double-blind, parallel arm clinical trial	Monocentric	100	Hospitalized	NA	Curcumin–piperine could alleviate coronavirus disease’s clinical symptoms, duration, severity, and inflammatory mediators	NA
Miryan et al., *Trail_propolis*, 2020 [28]	Iran	Second wave-ongoing (ending March 2021)	Double-blind, Placebo-controlled, parallel arm, randomized phase II clinical trial	Monocentric	80	Hospitalized	NA	Propolis supplementation changes coronavirus disease’s clinical symptoms, duration, and severity	NA

Abbreviations: NA, not available; BMI, Body Mass Index; CONUT, Controlling Nutritional Status; IMV, invasive mechanical ventilation; H1N1, Hemagglutinin Type 1 and Neuraminidase Type 1; LOS, length of hospital stay; ARDS, acute respiratory distress syndrome; GNRI, Geriatric Nutritional Risk Index; NRS 2002, Nutritional Risk Score 2002; MNA-sf, Mini Nutritional Assessment (short form); NRI, Nutritional Risk Index; GLIM, Global Leadership Initiative on Malnutrition.

## Data Availability

Not applicable.
